# Lifestyle-Associated Biomarkers of Cellular and Whole-Organism Aging: A Practical Roadmap for Human Geroscience Trials

**DOI:** 10.3390/biom16101504

**Published:** 2026-10-15

**Authors:** Stephen C. Rose, Albert A. Rizvanov, Donald D. Haines, Fred M. Cowan, Robert H. Silverstein, Fadia F. Mahmoud, Valeriya V. Solovyeva, Kristina V. Kitaeva, Seo Hyo-Seok, Arpad Tosaki

**Affiliations:** 1 Department of Science and Engineering, College of Nanotechnology, SUNY Albany, Albany, NY 12222, USA; chris@geronimo.life; 2 Institute of Fundamental Medicine and Biology, Kazan (Volga Region) Federal University, 420008 Kazan, Russia; solovyovavv@gmail.com (V.V.S.); kitaeva.kristina.v@gmail.com (K.V.K.); 3 Division of Medical and Biological Sciences, Tatarstan Academy of Sciences, 420111 Kazan, Russia; 4 Advanced Biotherapeutics Ltd., 20–22, Wenlock Road, London N1 7GU, UK; donalbain88@yahoo.com (D.D.H.); fadia.frcpath@gmail.com (F.F.M.); 5 Uppsala Inc., 67 Shady Brook Drive, Colora, MD 21917, USA; fred.cowan@zoominternet.net; 6 Preventive Medicine Center (PMC), 1000 Asylum Avenue, #2109, Hartford, CT 06105, USA; h.robert.silverstein@gmail.com; 7 Pyunkang Korean Medicine Hospital, 21 Seon-ro 1-gil, Seocho-gu, Seoul 06733, Republic of Korea; ha8251@gmail.com; 8 Department Pharmacology, Faculty of Pharmacy, University of Debrecen, Nagyerdei krt. 98, 4032 Debrecen, Hungary; 9 HUN-REN-UD Pharmamodul Research Group, Faculty of Pharmacy, University of Debrecen, Nagyerdei krt. 98, 4032 Debrecen, Hungary

**Keywords:** biological aging, geroscience, cellular senescence, inflammaging, epigenetic clocks, lifestyle intervention, biomarkers, healthspan, oxidative stress, mitochondrial dysfunction

## Abstract

Aging is a multidimensional biological process that is not adequately captured by chronological age alone. Geroscience—the study of biological mechanisms that drive age-related functional decline and disease susceptibility—therefore requires biomarker frameworks that can identify biological aging trajectories and assess whether lifestyle-associated interventions meaningfully influence aging-related pathways. In this review, we propose a practical, mechanism-based framework for studying lifestyle-associated effects on cellular and whole-organism aging in humans. We examine major biomarker domains, including DNA-methylation-based epigenetic aging clocks, cellular senescence-associated measures, senescence-associated secretory phenotype factors, inflammaging mediators, oxidative stress indicators, mitochondrial function, metabolic and nutrient-sensing pathways, proteomic and metabolomic signatures, microbiome-associated markers, and functional measures of frailty, mobility, cognition, and resilience. We also discuss criteria for biomarker selection in exploratory and multicenter trials, emphasizing biological relevance, analytical validity, responsiveness to intervention, feasibility, reproducibility, and clinical interpretability. Finally, we outline a practical study-design roadmap integrating biospecimen collection, repeated assessments, core and exploratory biomarker panels, and functional endpoints. A biomarker-guided geroscience strategy may provide a practical route for connecting mechanistic aging biology with clinically relevant human studies designed to preserve healthspan and functional independence.

## 1. Introduction: From Anti-Aging Claims to Measurable Geroscience

Human aging is associated with progressive loss of physiological reserve, increased vulnerability to stressors, and rising risk of chronic disease, frailty, disability, and mortality. Yet chronological age remains an imprecise surrogate for the biological processes that underlie this decline. Individuals of the same age may differ substantially in physical performance, metabolic health, immune competence, cognition, and resilience to disease. This heterogeneity has intensified interest in biomarkers that can better characterize biological age and reveal whether an intervention is modifying aging-related pathways rather than merely changing isolated clinical variables [1,2,3].

The term “anti-aging” remains widely used in public discourse, but it is scientifically imprecise and often associated with claims that exceed the available evidence. In contrast, geroscience seeks to understand the biological mechanisms that drive age-related functional decline and disease susceptibility and to identify interventions that preserve healthspan, defined here as the period of life spent in good health and functional independence. Within this framework, lifestyle-associated exposures such as exercise, diet quality, sleep regularity, circadian alignment, and stress regulation are attractive because they are broadly applicable, comparatively low risk, and directly relevant to preventive medicine. However, without robust biomarkers, claims regarding their effects on aging biology remain difficult to compare across studies and difficult to translate into trial design or clinical practice.

Biomarkers are particularly important in human aging studies because aging unfolds over decades, whereas most interventional trials are conducted over weeks to months. Molecular and functional indicators may therefore provide earlier evidence of biological response than hard clinical outcomes alone. At the same time, the complexity of aging means that no single marker is likely to be sufficient. A practical geroscience framework must integrate mechanistically informative biomarkers with clinically interpretable functional endpoints [3].

The aim of this review is to develop a translational biomarker framework for evaluating lifestyle-associated modulation of cellular and whole-organism aging in human geroscience studies. We focus on biomarker domains that are biologically relevant, feasible in human research, and potentially responsive to lifestyle intervention, while also identifying major methodological challenges that must be addressed before such markers can support multicenter trials and clinical translation.

This narrative review proposes a trial-design framework rather than a validated clinical guideline.

## 2. Conceptual Framework: Biological Aging Across Multiple Levels

### 2.1. Cellular Senescence as One Mechanism of Biological Aging

**Figure 1:**
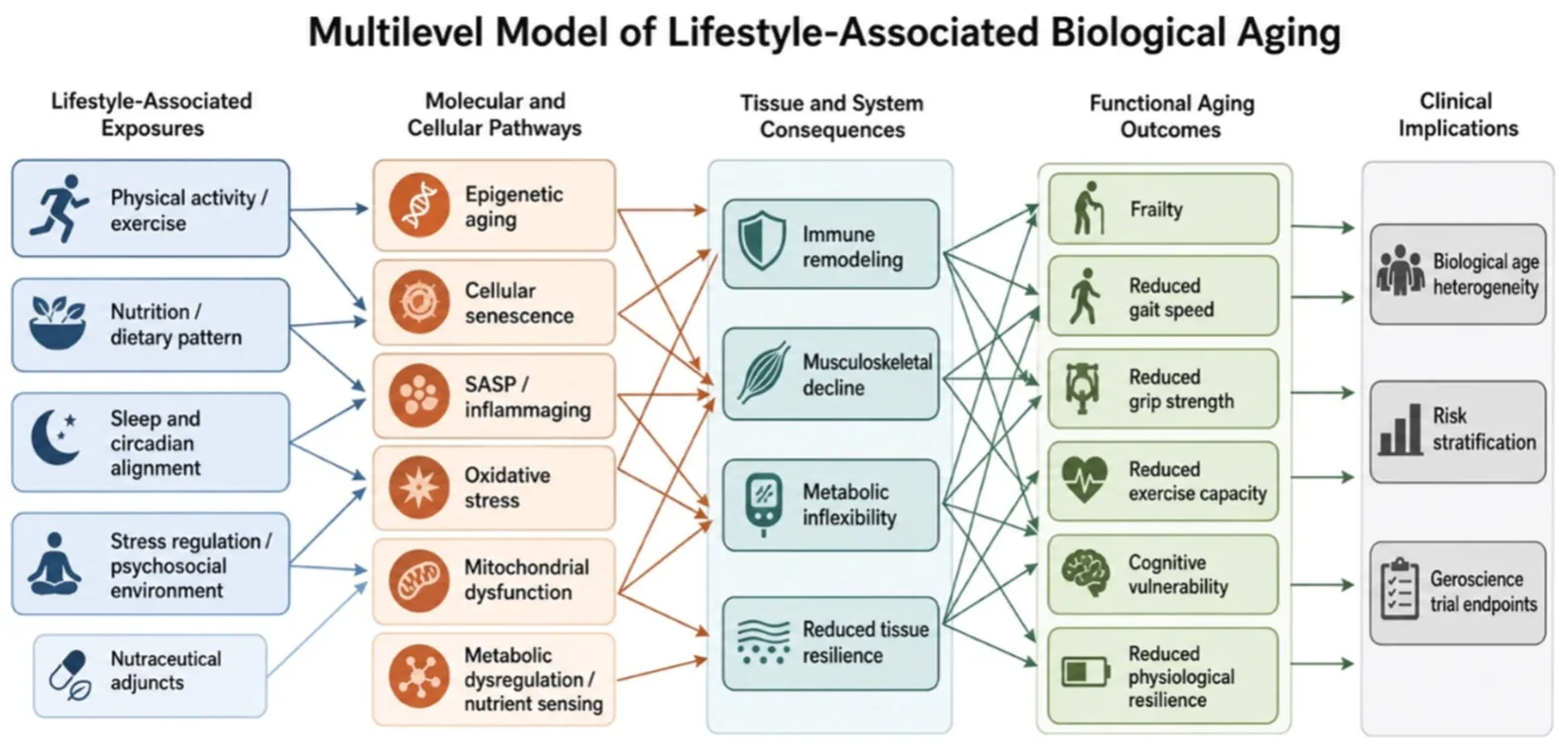
Conceptual framework linking cellular and systemic mechanisms of biological aging with measurable biomarker domains and whole-organism functional decline. The pathways shown are illustrative rather than exhaustive. Arrows indicate proposed biological relationships, not proof that each exposure changes each endpoint; cellular senescence is one component of biological aging.

Cellular senescence is a stress-responsive state characterized by durable cell-cycle arrest, altered metabolism, apoptosis resistance, and, in many contexts, a senescence-associated secretory phenotype (SASP). Commonly used senescence-associated readouts include p16^INK4a^, p21^CIP1/WAF1^, senescence-associated β-galactosidase activity, and SASP factors such as interleukin (IL)-6, IL-8, chemokines, and matrix-remodeling proteases [4,5]. This multilevel relationship between cellular aging mechanisms, systemic biomarker domains, and whole-organism functional decline is summarized in Figure 1.

Senescence can be beneficial in tissue repair and tumor suppression, but persistent accumulation of senescent cells is associated with chronic inflammation, impaired regeneration, and tissue dysfunction. Vascular and cardiometabolic tissues are particularly relevant because age-related risk factors such as hyperlipidemia, atherosclerosis, insulin resistance, and diabetes intersect with inflammatory and senescence-associated pathways [5,6,7].

This phenomenon may at least partly account for the observation that risk factors leading to cardiovascular disease, including hyperlipidemia, atherosclerosis, heart failure, insulin resistance, and type 2 diabetes, are major contributors to mortality with advancing age [7]. Indeed, the deleterious effect of senescent cell products on healthy tissues may contribute to age-associated physical deterioration [5,8]. Nevertheless, senescence has been difficult to quantify directly. No single marker uniquely identifies senescent burden across tissues and contexts. Accordingly, translational studies often rely on composite or proxy approaches that include cell-cycle regulators, senescence-associated secretory phenotype factors, and functional indicators of tissue decline. This limitation reinforces the need for multi-domain biomarker panels rather than one-marker claims [5].

Cellular senescence should not be equated with biological aging or whole-organism decline. Other interacting processes include genomic instability, telomere attrition, epigenetic alterations, loss of proteostasis, impaired autophagy, mitochondrial dysfunction, altered nutrient sensing, stem-cell exhaustion, immune remodeling, and altered intercellular communication [9]. A trial should select the process most plausibly affected by its intervention rather than assign all biomarker changes to senescence.

### 2.2. Inflammaging and Immune Remodeling

Aging is commonly accompanied by chronic low-grade inflammation, often termed inflammaging. This state is associated with altered innate and adaptive immune function, increased cytokine signaling, and higher risk of frailty, metabolic dysfunction, cardiovascular disease, neurodegeneration, and mortality. Inflammatory mediators are appealing as trial biomarkers because they are relatively accessible in blood and often measurable with standardized immunoassays. Yet they are also highly context-sensitive, influenced by infection, adiposity, medications, psychosocial stress, sleep disruption, and recent physical activity [1,10,11,12].

### 2.3. Oxidative Stress and Mitochondrial Dysfunction

Oxidative stress and mitochondrial dysfunction occupy a central mechanistic position in aging biology because mitochondria integrate energy production, redox signaling, calcium handling, apoptosis, and inflammatory activation. Under physiological conditions, reactive oxygen species (ROS) act as signaling molecules; however, persistent or compartmentally dysregulated ROS production can damage lipids, proteins, and nucleic acids and can amplify inflammatory and senescence-associated signaling. In aging tissues, impaired mitochondrial quality control, altered calcium handling, and reduced oxidative capacity may create a feed-forward cycle in which mitochondrial dysfunction increases ROS generation, while ROS further disrupt mitochondrial and cellular function. These mechanisms are relevant to cardiovascular, neuromuscular, and neurodegenerative phenotypes of aging, but they are difficult to capture using a single circulating marker. Therefore, redox-related readouts should be interpreted together with inflammatory, metabolic, mitochondrial, and functional endpoints rather than treated as stand-alone indicators of biological age [13,14,15,16,17,18].

### 2.4. Epigenetic and Omics-Based Aging

Epigenetic clocks and related molecular-aging algorithms have emerged as some of the most widely discussed biomarkers of biological aging. DNAm PhenoAge estimates a clinical-phenotype-based aging construct, GrimAge/GrimAge2 are mortality-risk-trained methylation algorithms, and DunedinPACE estimates a pace-of-aging construct rather than age in years [19,20,21,22]. Proteomic and metabolomic signatures extend this concept by capturing higher-dimensional states of physiology and system-level dysregulation. These measures are promising because they may integrate information from multiple biological pathways, but they remain heterogeneous in interpretation, tissue specificity, and responsiveness to intervention [23,24].

### 2.5. Whole-Organism Aging and Functional Decline

The ultimate relevance of geroscience biomarkers depends on their relationship to whole-organism function. Frailty, sarcopenia, impaired mobility, reduced cardiorespiratory fitness, cognitive decline, and diminished physiological resilience reflect integrated aging across multiple systems. A useful biomarker framework must therefore bridge mechanistic biology and clinically meaningful outcomes rather than treating molecular change as sufficient [2,25].

Recent analysis using the artificial intelligence (AI)-based PathStAR framework identified nonlinear, tissue-specific structural aging patterns in post-mortem human samples [26]. These cross-sectional findings concern structural aging, not longitudinal change or direct measurement of cellular senescence, and do not establish that a blood marker represents aging in every organ. Their implications for baseline tissue state and intervention-response interpretation are considered in Section 6.1.

### 2.6. Proteostasis, Autophagy, Immune Aging, and Regenerative Reserve

These processes are important even when they do not have a validated circulating biomarker. Prespecified immune-cell phenotyping can describe naive/memory lymphocyte distributions and activation, but should not be labeled a direct measure of senescence. Autophagy and proteostasis require process-appropriate assays: a single abundance measurement of an autophagy-related protein does not establish flux, and cell- or tissue-based functional studies may be needed. Stem-cell exhaustion and regenerative reserve are likewise tissue-dependent and cannot be inferred from a fall in blood cytokines. Such measurements belong in targeted substudies rather than being replaced by nonspecific blood proxies [9,27,28].

In a small study of 24-month-old male mice, rapamycin treatment was associated with higher 12-week survival, increased renal Klotho expression, and autophagy-associated protein changes (higher LC3B-II and lower p62) in kidney and adipose tissue [29]. These tissue-based findings do not establish that Klotho mediates survival or validate these proteins as human intervention endpoints.

The major aging mechanisms discussed in this section and their corresponding biomarker domains are summarized in Table 1.

## 3. Criteria for Selecting Biomarkers for Healthspan-Oriented Human Geroscience Trials

Biomarker selection in geroscience trials should begin with a defined context of use rather than with an available assay. In practice, a biomarker may be used for discovery, trial enrichment, proof of mechanism, monitoring of biological response, safety assessment, risk stratification, or eventual clinical decision support. These uses require different levels of evidence. The U.S. Food and Drug Administration (FDA) and the National Institutes of Health (NIH) BEST (Biomarkers, EndpointS, and other Tools) framework and geroscience-specific biomarker proposals therefore support a fit-for-purpose approach: the marker, assay, sample type, timing of collection, and interpretation plan should match the specific question being asked in the study [36,57]. For lifestyle-associated or mixed-intervention studies, this principle is particularly important because changes in aging biology are expected to be modest, heterogeneous, and pathway-specific rather than uniform across all biomarker domains [58,59,60,61].

### 3.1. Biological Relevance

Biological relevance should be specified before the assay is selected. A candidate biomarker should reflect an aging-related process that is plausibly affected by the intervention and plausibly linked to loss of physiological reserve, such as senescent-cell accumulation and SASP activity, chronic low-grade inflammation, mitochondrial dysfunction, oxidative stress, altered nutrient sensing, epigenetic age acceleration, or multisystem functional decline [3,5,13,19,24,25,58]. For example, an exercise trial can justify inflammatory, metabolic, mitochondrial, and physical-performance readouts more directly than a broad untargeted omics panel. A trial focused on senescence biology should pair cell- or tissue-based evidence with an independent senescence-associated readout and a functional outcome; p16^INK4a^ or SASP-related blood measures alone are not complete proxies for tissue senescence [5,30,31,32,36]. Mechanistic plausibility is not sufficient to validate a biomarker, but it helps prevent the panel from becoming a catalogue of high-cost assays with unclear translational meaning.

### 3.2. Clinical Interpretability

A biomarker is most useful when its baseline value, trajectory, or treatment-related change can be interpreted in relation to outcomes that matter to patients and clinicians. In aging studies, such outcomes include frailty, gait speed, grip strength, short physical performance battery scores, cognition, multimorbidity, hospitalization, disability, and mortality [2,25,53,54,55,56,59,62]. The strongest candidates are not necessarily the most technically sophisticated; they are those that add interpretable information beyond chronological age and conventional risk factors. DNA methylation clocks, proteomic aging signatures, and metabolomic clocks may be valuable secondary or exploratory endpoints, but their interpretation should be anchored to functional or clinical measures because different clocks capture overlapping but non-identical constructs and may not respond uniformly to short interventions [19,20,23,24,58,60,61].

### 3.3. Responsiveness to Intervention

Responsiveness should be considered at the design stage, not after the data are collected. Some readouts may shift within weeks, including inflammatory markers, glucose–insulin indices, lipids, blood pressure, wearable-derived activity, and sleep measures. Other endpoints, such as DNA methylation algorithms, frailty indices, physical-performance measures, and high-dimensional omics signatures, may require longer follow-up or repeated sampling to detect a stable signal [1,30,54,59,62,63,64,65,66]. A practical protocol should therefore tier endpoints into short-term response markers, intermediate mechanistic markers, and longer-horizon outcome-associated markers. This distinction reduces the risk of interpreting a null result in a slowly responsive marker as evidence that the intervention had no biological effect.

### 3.4. Analytical Validity and Feasibility

Analytical validity must be judged for the specific assay, biospecimen, and site workflow, not for the biomarker name alone. The BEST framework separates analytical validation from clinical validation: the former asks whether the test reliably measures the intended analyte, whereas the latter asks whether the biomarker meaningfully measures or predicts the clinical concept in its intended context of use [57,60]. In multicenter studies, pre-analytical and operational factors can be as important as biological rationale. Fasting status, time of day, recent exercise, acute infection, medication changes, anticoagulant choice, processing time, storage temperature, freeze–thaw cycles, assay lot, batch effects, and platform drift should be standardized or explicitly recorded [10,11,37,48,49,51,61]. A marker that performs well in a specialized single-center laboratory may not be appropriate as a core endpoint if sample handling or data analysis cannot be harmonized across sites; such markers are better reserved for centralized assays or exploratory substudies.

### 3.5. Suitability for Exploratory Versus Confirmatory Trials

Exploratory and confirmatory trials should not use the same evidentiary threshold. Early-phase studies may include broad panels to identify responsive pathways and compare biomarker domains, provided that the primary hypotheses, sample-handling procedures, and statistical approach are prespecified. Confirmatory studies should use a smaller set of biomarkers with a clear context of use, reproducible measurement, established associations with aging-related outcomes, and plausible responsiveness to the intervention [36,57,59,60,61,62]. This distinction is especially important in geroscience, where measuring many promising markers can create an impression of mechanistic breadth without improving decision value. In practice, the most defensible design is a small standardized core panel, anchored by functional outcomes, with a clearly labeled exploratory tier for omics, microbiome, or specialized cellular assays.

## 4. Biomarker Domains for Lifestyle-Associated Aging Studies

### 4.1. Epigenetic Biological Age Biomarkers

DNA-methylation-based biomarkers are among the most developed molecular tools for assessing aging-related variation in humans. However, the clinical Phenotypic Age score and DNAm PhenoAge are different measures. Phenotypic Age combines chronological age with nine clinical biomarkers; DNAm PhenoAge was trained to estimate this score from methylation at 513 CpG sites. Its output is expressed on an age scale and reflects a morbidity- and mortality-related phenotype, rather than a direct count of cellular damage [22]. GrimAge uses methylation surrogates for selected plasma proteins and smoking exposure, together with age and sex, to predict mortality risk; GrimAge2 extends this model. These risk-trained outputs are also expressed on an age scale [20,21].

DunedinPACE was trained against longitudinal change in multiple physiological measures, using 143 CpG sites, and is scaled as a rate of biological change per chronological year. It is therefore a candidate secondary endpoint when the question concerns an intervention-associated difference in pace, whereas DNAm PhenoAge or a prespecified GrimAge version is more appropriate when the question concerns a phenotype- or mortality-risk-related aging signature [19,20,21,22]. Choice of clock, version, normalization, and blood-cell-composition adjustment should be fixed before analysis. Age-scale scores should be evaluated relative to chronological aging and a comparator, not interpreted as literal time gained.

Their limitations are equally important. These algorithms differ in training outcomes, CpG sets and weights, populations, and sensitivity to cell composition; they are not interchangeable measurements of one underlying quantity. Discordant changes may therefore reflect different constructs as well as measurement error. In a post hoc analysis of 777 older DO-HEALTH participants over three years, intervention-associated effects differed across the four clocks assessed [66]. This illustrates the need to prespecify the relevant construct rather than select a favorable clock after analysis. Assay cost, tissue specificity, batch effects, and uncertainty about causal interpretation remain relevant [24]. A lower DNAm age or pace estimate is not, by itself, evidence of rejuvenation, reduced tissue senescence, or extended lifespan [58,60].

### 4.2. Cellular Senescence Biomarkers

Candidate senescence-associated readouts include p16^INK4a^, p21^CIP1/WAF1^, p53-pathway activity, senescence-associated β-galactosidase in cell- or tissue-based settings, and proteins linked to the SASP. None is individually diagnostic: p16^INK4a^ and p21^CIP1/WAF1^ can be expressed in non-senescent contexts, some senescent cells do not strongly express them, and lysosomal activity or cytokine secretion alone does not establish durable arrest [5,27]. p16^INK4a^ expression in peripheral blood T lymphocytes is an accessible aging-associated measure, but it is not a validated quantitative surrogate for senescent-cell burden in visceral adipose tissue, muscle, vascular endothelium, or brain [27,32]. Blood–tissue relationships depend on the cell population, tissue, disease, and senescence inducer; they should not be assumed to be concordant.

Four levels of evidence should be distinguished: (i) cellular senescence supported by several complementary features in an identified cell or tissue population; (ii) senescence-associated readouts, such as p16^INK4a^ expression or selected SASP proteins; (iii) systemic inflammatory markers, such as hsCRP or circulating IL-6; and (iv) integrative aging-related predictors, such as DNAm algorithms. A marker can contribute to more than one level, but an association does not confer specificity. Where feasible, combine cell-cycle-arrest evidence with an independent damage/lysosomal or structural readout in the same population, and add a SASP-related measure and a functional endpoint [5,27,31]. PBMC studies should standardize cell isolation and record cell composition. If only blood proxies are available, the conclusion should be limited to a change in circulating senescence-associated signals, not removal of senescent cells from tissues.

### 4.3. SASP and Inflammaging Biomarkers

Circulating inflammatory and SASP-related factors are among the most pragmatic biomarkers for human studies. Common candidates include high-sensitivity C-reactive protein, IL-6, tumor necrosis factor (TNF)-α, IL-1β, IL-8, monocyte chemoattractant protein (MCP)-1/C-C motif chemokine ligand (CCL)-2, C-X-C motif chemokine ligand (CXCL)-10, growth differentiation factor 15 (GDF-15), plasminogen activator inhibitor 1 (PAI-1), fibrinogen, and ferritin. These markers may help link cellular stress and immune remodeling to systemic consequences of aging. GDF-15 is of particular interest as an integrated stress-associated biomarker linked to mitochondrial stress, cellular senescence-associated signaling, multimorbidity, and impaired physical function. It is not specific for mitochondrial damage or senescent-cell burden; renal function, cardiometabolic disease, malignancy, and medication exposure may affect its concentration [1,31,33,34,35].

Their practicality should not be confused with specificity. Acute infection, obesity, smoking, medication exposure, psychosocial stress, poor sleep, and recent exercise can all alter inflammatory readouts. Repeated measurements, standardized collection windows, and contextual covariate capture are therefore essential [1,10,11].

### 4.4. Oxidative Stress and Redox Biomarkers

Potential redox-related biomarkers include 8-hydroxy-2′-deoxyguanosine, F2-isoprostanes, malondialdehyde, protein carbonyls, oxidized-to-reduced glutathione ratios, and selected markers of endogenous antioxidant defense. These readouts are biologically plausible because ROS and redox signaling participate in mitochondrial dysfunction, inflammatory activation, calcium dysregulation, senescence, and regulated cell-death pathways. However, redox biomarkers are among the most pre-analytically sensitive markers in aging studies: results may depend on sample matrix, processing time, storage conditions, assay platform, recent exercise, acute infection, medication exposure, and supplement use. For multicenter geroscience trials, prioritize chemically specific, matrix-validated measurements, such as F2-isoprostanes by isotope-dilution GC-MS or LC-MS/MS, or urinary 8-hydroxy-2′-deoxyguanosine by validated LC-MS/MS [38,39]. Nonspecific colorimetric total antioxidant capacity assays, unvalidated malondialdehyde kits, and thiobarbituric-acid-reactive-substances assays should not be primary evidence of reduced oxidative damage. Mass spectrometry does not remove the need to control processing delays, ex vivo oxidation, storage, and freeze–thaw cycles. Urinary measurements also require a prespecified dilution-adjustment method and consideration of renal function. Expanded redox panels should remain exploratory unless assays are centralized and supported by strict standard operating procedures. Redox biomarkers should therefore be used as part of an integrated panel rather than as independent evidence of slowed biological aging [14,15,16,17,18,37].

### 4.5. Mitochondrial Function Biomarkers

Mitochondrial biomarkers describe different biological properties. mtDNA copy number estimates mitochondrial genome abundance per cell or relative to nuclear DNA; it is not equivalent to mitochondrial mass, ATP production, or respiratory capacity. Mutation burden concerns sequence alterations, deletions are structural losses of mtDNA, and heteroplasmy is the proportion of different mitochondrial sequence variants within a sample. Their functional consequences depend on the variant, its abundance, and the affected cell or tissue [40,42]. Age-related mtDNA changes are not uniform: copy number may rise, fall, or remain stable across tissues, while damaging variants or deletions may accumulate in selected cell populations. A higher copy number may reflect compensatory replication rather than improved function [40,42].

Whole-blood mtDNA measurements are strongly influenced by leukocyte composition and platelet contamination, because platelets contain mitochondria but no nuclear genome. PBMC respiration instead assesses oxygen-consumption characteristics in the sampled immune-cell mixture and requires standardized viability, cell counts, substrate/inhibitor conditions, processing times, and, where used, cryopreservation [28]. Neither readout can be assumed to represent skeletal-muscle mitochondrial function. For a muscle-focused trial, tissue-specific respirometry or 31P-MRS may be more informative where feasible. Mitochondrial content measures, respiratory function, and mtDNA sequence integrity should therefore be reported separately rather than combined under an unspecified mitochondrial-health label [28,40,41,42].

For multicenter human studies, mitochondrial measurements are best organized as mechanism-specific modules: pair a functional assay with an independent content-related measure, such as citrate synthase activity or mtDNA copy number in the same defined cell population. Report both per-cell function and any content-normalized result. Mutation/deletion or heteroplasmy assays should be added only for a prespecified genomic question; a short lifestyle intervention need not change these relatively distinct endpoints [28,42].

### 4.6. Metabolic and Nutrient-Sensing Biomarkers

Markers such as fasting glucose, insulin, homeostatic model assessment of insulin resistance (HOMA-IR), glycated hemoglobin (HbA1c), lipid panels, insulin-like growth factor 1 (IGF-1), adiponectin, leptin, uric acid, and selected amino acid or acylcarnitine profiles provide clinically interpretable readouts of pathways implicated in aging biology. These measures connect nutrient sensing, metabolic flexibility, insulin signaling, and systemic disease risk. They are also among the most feasible and standardized endpoints available for large human studies. Although they are not specific to aging, they are often responsive to lifestyle modification and clinically meaningful, making them valuable components of a translational biomarker framework [43,44,45].

### 4.7. Proteomic and Metabolomic Aging Signatures

High-dimensional proteomic and metabolomic platforms may capture integrated physiological states and reveal latent aging-related patterns not visible through conventional single-analyte testing. Proteomic aging clocks, inflammatory protein panels, extracellular matrix remodeling signatures, and metabolomic risk profiles may all contribute to a more nuanced picture of biological aging [23,46,47,50,52].

These tools are promising but should be approached cautiously in confirmatory settings. Platform comparability, batch effects, data normalization, model portability, and reproducibility remain major issues. At present, such markers are best positioned as exploratory or secondary endpoints unless strong analytical and prognostic validation already exists [47,48,49,51,52].

### 4.8. Microbiome-Associated Aging Biomarkers

The gut microbiome has been linked to inflammation, frailty, metabolic function, immune signaling, and healthy aging trajectories. Candidate readouts include diversity measures, short-chain fatty acid profiles, gut barrier markers, and broader compositional or functional microbiome signatures. This domain is conceptually relevant, but human findings remain heterogeneous, and standardization challenges are substantial. Microbiome markers may therefore be useful as an exploratory domain rather than a core component of a primary geroscience score [67,68]. In the one-year NU-AGE dietary intervention, diet-responsive microbiome patterns were associated with frailty-related and inflammatory measures; these findings support exploratory microbiome assessment but do not validate a universal microbiome aging score or establish lifespan extension [67].

### 4.9. Functional and Clinical Outcome Assessments of Whole-Organism Aging

Functional endpoints should occupy a central rather than peripheral place in human geroscience trials. Frailty index, gait speed, grip strength, short physical performance battery, six-minute walk distance, balance testing, body composition, sleep metrics, cognitive testing, heart rate variability, and cardiorespiratory fitness provide complementary information about whole-organism aging. Systematic reviews and meta-analyses show that gait speed, grip strength, and broader physical capability measures predict mortality, disability, falls, and hospitalization, supporting their inclusion as anchor outcomes in geroscience studies. An analysis of a physical-activity trial also suggests that circulating senescence biomarkers predict incident mobility disability and may be modifiable with higher levels of physical activity. These outcomes help prevent overinterpretation of molecular changes whose clinical significance remains uncertain [2,25,53,54,55,56]. Under the BEST terminology, performance-based measures such as gait speed, grip strength, and the SPPB are clinical outcome assessments, not molecular biomarkers [57].

## 5. Lifestyle Interventions as Tools to Probe and Mitigate Effects of Biological Aging

Lifestyle interventions should not be presented as proven anti-aging therapies. Rather, they should be framed as testable modulators of aging-related pathways whose biological effects can be evaluated using standardized biomarker systems. Table 2 links each intervention to a target process, main readout, complementary measurement, functional outcome, and illustrative response window.

### 5.1. Physical Activity and Exercise

Exercise is one of the most plausible and measurable lifestyle modulators of aging biology. Aerobic training, resistance training, and combined programs may influence inflammatory tone, mitochondrial capacity, insulin sensitivity, body composition, muscle function, and frailty. Because exercise interventions often produce measurable physiological changes within months, they provide a useful test case for biomarker responsiveness in geroscience trials. Human intervention studies now support reductions in circulating senescence-related biomarkers and improvements in physical function after structured exercise, while systematic reviews and meta-analyses indicate broader though heterogeneous effects on inflammatory pathways in older adults. Recent intervention studies also support effects on mitochondrial capacity and redox balance, although protocols and biomarker depth vary substantially across trials [41,63,64,65,69,70].

Exercise can engage AMPK-related energy sensing, mitochondrial biogenesis, autophagy, insulin signaling, and inflammatory regulation, depending on the protocol [9]. For a mitochondrial hypothesis, Table 2 pairs respiratory capacity with a content-related measure in the same sample. Changes in hsCRP or fitness alone do not demonstrate AMPK activation or autophagic flux [28].

### 5.2. Nutrition and Dietary Patterns

Dietary quality, caloric balance, protein adequacy, meal timing, and micronutrient status are all relevant to aging-related physiology. Mediterranean-style dietary patterns, caloric restriction, time-restricted eating, and strategies targeting glycemic control may affect metabolic biomarkers, inflammatory mediators, and potentially epigenetic or proteomic aging signatures. However, dietary interventions vary greatly in adherence, duration, and mechanistic specificity, making careful exposure characterization essential [30,45,71]. These interventions may involve nutrient-sensing (AMPK–mTOR), autophagy, or metabolic-flexibility pathways, but weight loss alone does not establish pathway engagement [9].

Numerous prospective studies associate dietary patterns with healthier aging, and randomized dietary trials provide evidence for specific clinical benefits. For example, the CORDIOPREV trial compared Mediterranean and low-fat diets in patients with established coronary heart disease over seven years and found fewer major cardiovascular events with the Mediterranean diet [71]. This is evidence for a defined clinical outcome in a defined population, not direct proof of lifespan extension. Large cohort studies also associate higher-quality, plant-rich dietary patterns with healthier aging and lower cardiovascular risk, although residual confounding limits causal interpretation [80,81]. Direct randomized evidence that dietary patterns extend human lifespan remains limited; intervention duration, adherence, nutritional adequacy, and changes in adiposity should be considered when interpreting biomarker responses.

Caloric restriction reduces energy intake, whereas time-restricted eating changes the daily eating window and may not reduce intake. Findings on senescence-associated measures after caloric restriction [30] should not be generalized to time-restricted eating. A small controlled early time-restricted-feeding study in men with prediabetes found metabolic effects without weight loss, not evidence of slower biological aging [45].

### 5.3. Sleep and Circadian Biology

Sleep quality, sleep duration, and circadian alignment affect immune regulation, metabolic control, cognition, and stress physiology. Because sleep disruption is common in midlife and older age, this domain is highly relevant yet often underrepresented in biomarker-driven geroscience research. Reviews of normal aging and circadian biology indicate that age-related changes in sleep timing, continuity, and circadian regulation may themselves be informative readouts of systemic aging processes. Sleep-focused studies may benefit from combining inflammatory and metabolic markers with objective sleep measures and wearable-derived behavioral data [72,73].

### 5.4. Stress, Autonomic Regulation, and Psychosocial Factors

Chronic psychosocial stress, dysregulated cortisol rhythms, loneliness, depression, and autonomic imbalance may influence inflammation, metabolic health, immune function, and resilience. Heart rate variability and diurnal cortisol profiles are potential adjuncts in this domain. Mind–body interventions can be incorporated, but they should be evaluated as measurable exposures with testable biological effects rather than as vague wellness claims. Recent randomized trials in older adults show that mindfulness-based stress reduction can alter proinflammatory transcriptional regulation and selected immune outcomes, even when effects on circulating inflammatory proteins are mixed [74,75].

## 6. Baseline State and Pathway-Level Measurement in Geroscience Trials

### 6.1. Baseline Injury as a Modifier of Biomarker Responsiveness

Biomarker response to an intervention may depend substantially on the participant’s biological starting state. This is particularly important in geroscience trials, where older adults commonly enter studies with heterogeneous burdens of chronic disease, previous treatment, environmental exposure, and established organ injury. Biomarkers intended to measure intervention effects should therefore be evaluated for responsiveness, reproducibility, tissue relevance, and clinical interpretability in the population under study [36,61].

Pre-existing structural injury may constrain the range and rate over which some measures can change during a practical trial interval. A systemic inflammatory or metabolic biomarker may respond while an organ-specific functional measure changes little because established structural damage changes more slowly. Conversely, persistent abnormality of a circulating marker may reflect ongoing organ-specific pathology rather than accelerated whole-organism aging. Discordance among biomarkers should therefore not automatically be interpreted as either intervention failure or evidence of biological rejuvenation.

Where substantial previous injury is plausible, baseline burden should be characterized using measures appropriate to the target organ and intervention. These may include pulmonary-function testing, renal function and albuminuria, cardiovascular imaging or functional indices, musculoskeletal imaging or performance measures, and validated assessments of frailty or multimorbidity. When repeatable tissue-level assays are unavailable, organ-specific structural or functional measurements provide a practical orthogonal estimate of tissue state.

To illustrate how persistent tissue injury can constrain interpretation of generalized aging biomarkers, long-term sulfur-mustard injury provides an extreme but informative boundary-condition model. Approximately 34,000 Iranians known to have survived sulfur-mustard exposure for more than a decade were evaluated for late health effects, with pulmonary, ocular, and cutaneous complications documented in substantial subsets [82]. A subsequent bronchial-biopsy study examined p53 immunoreactivity in relation to exposure and smoking [83].

Long-term follow-up further illustrates the potential discordance between circulating biomarkers and organ-level disease. In the Sardasht-Iran Cohort Study, 348 sulfur-mustard-exposed participants and 120 controls were evaluated approximately two decades after exposure. Circulating IL-6 and IL-8 concentrations were lower in exposed participants than in controls, while IL-8 showed no significant relationship to respiratory symptoms, pulmonary physical findings, or spirometric parameters; IL-6 was associated with wheezing [84]. The investigators concluded overall that serum IL-6 and IL-8 did not adequately reflect the severity of chronic pulmonary involvement. Thus, a circulating inflammatory marker may characterize biological state or exposure without reliably grading organ-specific injury.

A related intervention study illustrates differing short-term response kinetics. In a four-week randomized placebo-controlled trial enrolling 89 men with chronic sulfur-mustard pulmonary complications, 78 participants completed follow-up. Curcuminoids altered multiple circulating inflammatory mediators, whereas forced expiratory volume in one second (FEV1) and forced vital capacity (FVC) did not differ between groups, and FEV1/FVC showed a selective improvement [85]. The short treatment interval and isolated ratio change preclude inference of generalized pulmonary recovery; the study is used here only to illustrate that systemic biomarkers and organ-level functional measures may respond differently over the same interval. These studies do not establish that fixed structural injury caused the observed discordance.

The methodological issue is not specific to military exposure. Older participants in lifestyle and geroscience trials may carry persistent or slowly reversible injury arising from chronic pulmonary disease, cardiovascular disease, previous cancer treatment, renal disease, severe infection, or other accumulated insults. Baseline tissue state may therefore modify the measurable response to an intervention and should be considered during participant characterization, stratification, adjustment, and interpretation.

Recent tissue-wide structural analysis reinforces the importance of tissue specificity. Yadav et al. used the AI-based PathStAR framework to analyze 25,306 post-mortem biopsies from 40 tissues in 970 donors aged 21–70 years and identified distinct nonlinear age-associated structural patterns across tissues [26]. Periods of greater structural deterioration were associated with increased inflammation and reduced energy production, repair, and quality-control processes. These patterns were inferred cross-sectionally across different donors rather than observed longitudinally within individuals, and PathStAR is based on post-mortem histopathology rather than a repeatable clinical-trial assay. Its relevance here is therefore interpretive: a generalized circulating or composite measure of biological aging should not automatically be assumed to represent equivalent aging or responsiveness in every organ.

For intervention studies, these considerations support the following sequence:

Baseline injury state → targeted mechanism/pathway → response biomarker → orthogonal organ-specific or functional measure → prespecified direction and expected time course of change.

This extends the biomarker roadmap in Table 2 by recognizing baseline tissue state as a potential modifier of both the magnitude and kinetics of intervention response.

### 6.2. Cross-Condition Validation of Pathway-Level Effects

When an intervention is proposed to act on a shared host pathway rather than on a disease-specific lesion, concordant benefit across biologically distinct conditions becomes a testable prediction. Geroscience applies this logic in the Targeting Aging with Metformin (TAME) framework, which asks whether an intervention directed at aging biology can delay multiple age-related diseases and functional decline rather than merely affect one clinical condition [36].

A related discrimination criterion was developed in the Multi-Threat Medical Countermeasure (MTMC) literature for biologically distinct toxic injuries: efficacy of a countermeasure across different threats can strengthen the inference that it acts on shared biochemical signaling pathways, molecular mediators, or cellular processes rather than solely on an insult-specific mechanism [86].

While the conceptual relationship between MTMC, oxidative stress, chronic inflammation, aging, healthspan, and AI-assisted analysis has been examined previously [87], the present review addresses the measurement criteria needed to test such pathway-level effects in human geroscience.

Cross-condition concordance is a validity heuristic rather than proof. The same intervention could affect different diseases through different mechanisms; consequently, concordant clinical outcomes are most informative when accompanied by movement in a mechanistically appropriate shared-pathway biomarker and meaningful functional endpoints.

Cross-condition evidence may be obtained within a multimorbid population or across separate disease-specific studies, but the two designs have different strengths and limitations. Within-person or within-cohort comparisons hold the intervention environment relatively constant but complicate attribution among coexisting diseases; comparisons across separate trials permit cleaner disease-specific attribution but introduce differences in populations, assays, treatment intervals, and baseline injury burden. These sources of heterogeneity should be considered when interpreting apparent concordance or discordance.

Recent experimental geroscience is consistent with the value of examining such convergence. Austad, Kaeberlein, and Miller describe candidate aging-rate indicators that change across several genetic, dietary, and pharmacological interventions associated with increased lifespan in mice [88]. Shared changes across successful interventions may identify pathway-level responses worthy of further study. However, convergence does not demonstrate that these changes mediate longevity; they may represent causal mechanisms, downstream consequences, or correlated effects of altered growth, energy metabolism, or other biological processes. These indicators are predominantly mouse-derived, and lifespan extension is not equivalent to demonstrated improvement in human healthspan.

The practical implication for a geroscience biomarker roadmap is testable: when an intervention is proposed to act on an aging mechanism rather than on a single disease, evidence should be sought across multiple clinically distinct outcomes, accompanied where possible by concordant movement in biomarkers of the hypothesized shared pathway and by meaningful functional endpoints. Consistency across conditions strengthens—but does not independently establish—the case for pathway-level action.

### 6.3. Interaction of Baseline State and Cross-Condition Validation

Baseline injury state and cross-condition validation are interdependent. An intervention may genuinely modify a shared biological pathway yet produce an attenuated or absent organ-level response in the condition carrying the greatest fixed or slowly reversible structural injury. Such a result could create a false-negative cross-condition test, making genuine pathway-level action appear disease-specific. Conversely, unequal baseline injury burdens across the conditions being compared can generate apparent biological discordance even when underlying pathway responses are similar.

Cross-condition comparisons should therefore characterize baseline injury burden in each relevant organ or condition and interpret response in relation to the magnitude and time course of change that was biologically available. Concordance or discordance should be judged not simply by whether biomarkers move, but by what each measure represents, the starting tissue state, and the response kinetics expected for that biological domain.

Thus, a rigorous pathway-level test requires both cross-condition evidence and an accounting of the baseline biological constraints under which each condition was capable of responding.

## 7. A Practical Biomarker Framework for Multicenter Human Geroscience Trials

Multicenter studies need a deliberately restricted common protocol. Their purpose is not to measure every plausible hallmark of aging at every site, but to obtain a reproducible set of molecular and functional data that can answer a prespecified question. Before recruitment begins, investigators should define the intended use of each marker—participant characterization, proof of mechanism, monitoring of biological response, safety assessment, or prediction—and link that use to the assay platform, specimen type, sampling schedule, and statistical role [36,57,59,60].

### 7.1. Biospecimen Collection and Pre-Analytical Control

Blood should form the core of biospecimen collection because it supports routine clinical chemistry, inflammatory-protein measurements, DNA methylation assays, metabolomics, proteomics, and selected cellular analyses. A practical minimum is ethylenediaminetetraacetic acid (EDTA) whole blood, plasma, and serum; urine can be added for prespecified redox or metabolite assays. PBMCs should be collected only when every site can follow the same isolation, cryopreservation, shipment, and viable-recovery procedure. Stool and saliva should be optional rather than default specimens and should be collected only when microbiome or salivary endpoints are specified in the protocol [28,89,90].

All sites should work from a single specimen manual and use common collection tubes, labels, aliquot volumes, and shipping procedures. The protocol should specify fasting status, collection time, recent vigorous exercise, acute illness, smoking and alcohol exposure, changes in medications or supplements, order of draw, time to centrifugation, processing temperature, storage temperature, and the permitted number of freeze–thaw cycles. These variables should be recorded for each visit rather than inferred from local laboratory practice. A pilot collection before enrollment is advisable to confirm processing times, specimen yield, PBMC viability where relevant, and cross-site comparability. Samples intended for omics or batched protein assays should be balanced across assay plates by treatment arm, site, sex, and visit, with pooled quality-control material and blinded technical replicates. Biospecimen reporting for improved study quality (BRISQ)- and standard preanalytical code (SPREC)-compatible documentation provides an established basis for these procedures [10,11,48,49,51,89,90].

### 7.2. Timing of Repeated Assessments

Sampling frequency should be derived from the expected kinetics of the intervention and the biomarker, rather than copied from a generic timetable. For example, a 12-month lifestyle trial could include baseline, an early visit at 4–6 weeks, an intermediate visit at 3 or 6 months, and an end-of-intervention visit at 12 months. A post-intervention visit is justified when persistence or reversibility of the response is a prespecified objective. For markers with substantial within-person variability, duplicate baseline measurements separated by several days or weeks should be considered when the additional burden is acceptable.

Inflammatory, metabolic, adherence, and safety measures can often be assessed early, whereas sustained changes in physical function, senescence-related measures, DNA methylation algorithms, and high-dimensional omics profiles may require longer follow-up. Repeat visits should be conducted under conditions that are as similar as possible to baseline, particularly with respect to time of day, fasting, and recent physical activity. The statistical analysis plan should identify the visit that tests the primary hypothesis and specify how repeated measurements will be modeled; collecting numerous time points without a defined contrast mainly increases multiplicity [36,57,60].

### 7.3. Core Measurements

The common core should be small enough to be completed using the same assay methods at all sites or in a central laboratory. Candidate blood-based measurements include high-sensitivity C-reactive protein, IL-6, GDF-15, and, when relevant, a soluble TNF receptor (sTNFR1 or sTNFR2), with the exact receptor and assay prespecified. Metabolic measures include fasting glucose, HbA1c, and a lipid profile; insulin and HOMA-IR can be added when fasting collection is reliably standardized [36,62]. The final subset should match the trial question rather than be mandatory for every study. These measures characterize systemic response and risk; they do not establish a specific aging mechanism. Table 2 provides intervention-specific examples of mechanism-focused and complementary measurements.

Functional anchors should include grip strength, usual gait speed, and the Short Physical Performance Battery. A frailty index or 6-min walk test can be added according to the population and trial objective. Under the FDA-NIH BEST terminology, these measures are clinical outcome assessments rather than biomarkers; this distinction should be retained even when molecular and functional outcomes are analyzed together [2,53,57,59].

One prespecified DNA methylation measure, selected by the construct distinctions in Section 4.1, may be included as a centrally analyzed secondary endpoint, but it should not be described as a validated surrogate for healthspan or lifespan. Measurements such as p16^INK4a^ expression, expanded senescence-associated secretory phenotype panels, F2-isoprostanes, 8-hydroxy-2′-deoxyguanosine, mitochondrial DNA copy number, and PBMC respirometry are better treated as mechanism-specific modules than as mandatory core measures, unless the intervention directly targets the relevant pathway and the assay workflow has been validated across sites [19,20,27,28,30,31,32,38,39,42,60,62]. The proposed division between common measurements and mechanism-specific modules is summarized in Table 3.

### 7.4. Extended Exploratory Measurements

High-dimensional or technically demanding assays belong in a prespecified exploratory tier. This tier may include proteomic or metabolomic aging signatures, expanded immune-cell phenotyping, senescence panels, microbiome profiles, nicotinamide adenine dinucleotide (oxidized form) (NAD)^+^/NAD (reduced form) (NADH)-related measures, PBMC bioenergetics, continuous glucose monitoring, heart-rate variability, and wearable-derived sleep and activity metrics [23,36,46,47,48,49,50,51,52,58,60,61,62,67,68].

Where possible, these analyses should be conducted in nested substudies with adequate sample size, centralized processing, batch-balanced sample placement, and an analysis plan that separates discovery from validation. If a new composite score is derived, its components, scaling, weights, direction of effect, and missing-data rules should be fixed before testing in an independent sample. Digital measures likewise require prespecification of device model, firmware, placement, minimum wear time, and signal-processing rules. A statistically significant exploratory result should not, by itself, be presented as evidence that an intervention slowed biological aging.

### 7.5. Endpoint Hierarchy and Target Population

Endpoint selection should begin with the intervention and the phase of development. An early proof-of-concept trial may use a proximal pharmacodynamic or response marker, or a functional measure, as the primary endpoint when it is closely linked to the proposed mechanism and is expected to change within the study period. Later-phase trials should place greater weight on outcomes that reflect how participants function or whether they experience clinically important events. Molecular measures can then serve as secondary endpoints of target engagement, pathway response, or effect heterogeneity [36,57,59,60,61].

No current composite biological-age score is a universally validated surrogate endpoint for healthspan or lifespan. An integrated geroscience score should therefore remain secondary or exploratory unless it has been validated for the same population, intervention, and context of use. When a composite is used, its components, weights, directionality, and handling of missing data must be prespecified; otherwise, an apparently favorable change can be driven by post hoc analytical choices [57,58,59,60,61].

The target population should also be coherent. A trial in pre-frail older adults and a prevention study in middle-aged adults with elevated cardiometabolic risk address different questions and should not be combined casually in a single primary analysis. Eligibility criteria should enrich for a measurable but potentially modifiable deficit without selecting participants who are too frail to tolerate the intervention. Randomization and analysis should account for site and, where appropriate, sex, age band, and baseline functional status. This focused approach is more likely to produce interpretable findings than a broadly defined and highly heterogeneous healthy-aging cohort [25,36,59,62].

## 8. Exploratory Adjuncts and Methodological Boundaries

Structured exercise, dietary optimization, sleep or circadian interventions, and stress-regulation programs remain the main intervention categories considered here. Selected nutraceutical add-on arms may be justified by prior mechanistic or human evidence. Pharmacological senotherapies are discussed only to clarify how senescence-directed hypotheses differ from general lifestyle effects, not as established healthspan- or lifespan-extension strategies.

### 8.1. Senotherapies: Cell Removal Versus Phenotype Modulation

Senotherapies are an umbrella category. Senolytics aim to preferentially eliminate senescent cells, whereas senomorphic approaches modify features such as the SASP without necessarily removing the cells; preventing entry into senescence is a separate intervention objective [5,91,92]. In animal models, senescent-cell targeting has improved selected functional outcomes and, in some studies, survival [93,94,95]. These findings do not establish human lifespan extension or a general indication for senolytic use.

The physiological context matters. Transient senescence can contribute to tissue repair and tumor suppression, so indiscriminate depletion may be undesirable [4,5,27]. Senescence and reversible quiescence are distinct states: removal of senescent cells does not inherently imply loss of healthy quiescent cells, and possible off-target toxicity must be evaluated separately. There is therefore no established rationale for assuming that senolysis requires mesenchymal stromal/stem-cell replacement. Where tissue sampling is feasible, a senescence-directed study could combine p16^INK4a^/p21^CIP1/WAF1^ with persistent DNA-damage or lysosomal features in identified cells, a separate SASP-related assay, and gait speed or SPPB [27,31]. A reduction in T-cell p16^INK4a^ expression or blood cytokines alone does not establish senolysis. Safety and loss of function must also be monitored.

### 8.2. Nutraceutical and Phytochemical Adjuncts

Nutraceuticals, phytochemicals, and compounds derived from traditional medical systems may be useful as hypothesis-generating adjuncts when linked to defined mechanisms, such as redox regulation, mitochondrial function, inflammatory signaling, or cellular stress responses. Their translational value depends on standardized composition, dose, bioavailability, formulation, safety, and clinically interpretable outcomes [76,78,79]. Traditional use or food-safety status does not establish efficacy or safety at a proposed trial dose. Such interventions should not be presented as proven life-extension therapies.

For example, heme oxygenase-1 (HO-1)-related redox responses and mitochondrial ROS production provide testable mechanistic hypotheses, but generalized antioxidant activity is not sufficient evidence. Sauchinone reduced mitochondrial ROS and senescence-related features in fibroblast models; this remains preclinical evidence [77]. A human adjunct study would need exposure and safety assessment, a validated pathway-relevant assay, an independent corroborating measure, and a clinically relevant outcome. The framework is intended to evaluate such hypotheses, not to endorse a particular formulation.

Pyunkang-Tang (PGT; also known as Pyunkang-hwan), a traditional Korean herbal preparation, has also been investigated in experimental respiratory disease. In rats, orally administered PGT reduced sulfur dioxide-induced airway mucin hypersecretion and attenuated bleomycin-induced pulmonary fibrosis, assessed by histopathology and lung hydroxyproline content [96]. These findings support further investigation of inflammatory airway injury and fibrosis, but do not establish clinical efficacy or reductions in systemic inflammaging, alveolar senescence, muscle wasting, or biological aging in humans.

For a future controlled PGT study, the Table 2 approach could pair a prespecified inflammatory biomarker with spirometry and six-minute walking performance, while documenting formulation, dose, safety, and background treatment. These are proposed trial measurements, not established PGT-responsive aging endpoints; any senescence-related claim would require separate cellular or tissue-level evidence.

Further, experimental studies of plant-derived compounds suggest potential for modulating cellular senescence and associated inflammatory pathways. Lycorine, an alkaloid found in Amaryllidaceae plants including *Lycoris radiata* (red spider lily), attenuated lipopolysaccharide-induced acute lung injury in mice, accompanied by reduced HMGB1/TLRs/NF-κB signaling [97]. In a separate study, lycorine hydrochloride directly bound to and activated the ubiquitin-activating enzyme UBA1, enhanced protein ubiquitination, and suppressed stress-induced premature cellular senescence and selected SASP factors in human fibroblast models [98]. These preclinical findings support further mechanistic investigation, but do not establish effects on whole-organism aging or clinical efficacy and safety. Results obtained with lycorine or its hydrochloride salt should not be extrapolated to PGT or other multicomponent herbal preparations.

### 8.3. Methodological Challenges

Several challenges complicate biomarker-guided geroscience research. First, many biomarkers show substantial intra-individual variability related to time of day, recent exercise, infection, diet, sleep, stress exposure, and medication use. Second, blood-based markers may not represent aging in all tissues equally. Third, many biomarkers are associated with aging outcomes without being established causal drivers. Fourth, composite scores are attractive but difficult to calibrate, validate, and interpret across populations [10,11,19,51]. Key practical sources of variability in multicenter biomarker studies and recommended mitigation strategies are summarized in Table 4.

These issues do not argue against biomarker use; rather, they argue for disciplined study design. Standardization of collection protocols, rigorous assay validation, repeated measurement, contextual covariate capture, and transparent analytic plans are prerequisites for interpretable findings. Multicenter studies should prioritize harmonization and reproducibility over maximal biomarker breadth.

## 9. Proposed Roadmap for Future Research

Future work may be organized in four overlapping phases. Phase 1 should emphasize discovery and feasibility, using broad panels to identify responsive and interpretable markers. Phase 2 should prioritize biomarkers that demonstrate reproducibility, intervention responsiveness, and clinical relevance. Phase 3 should test these prioritized biomarkers in multicenter validation studies across diverse populations and intervention types. Phase 4 should focus on clinical translation, including harmonized operating procedures, threshold development, and integration into preventive and geriatric assessment frameworks.

The field does not primarily need more broad claims about anti-aging interventions. It needs robust biomarker systems capable of distinguishing biological signal from noise and connecting mechanistic aging biology to outcomes that matter for human healthspan.

## 10. Conclusions

Recent advances in geroscience have shifted the field from broad anti-aging claims toward testable mechanisms, measurable biomarkers, and intervention studies designed to preserve function. Cellular senescence, inflammaging, mitochondrial dysfunction, metabolic dysregulation, and functional decline provide useful organizing domains, but major challenges remain in assay standardization, causal interpretation, tissue specificity, and clinical translation.

Aging is multidimensional and cannot be captured by chronological age or any single biomarker. Lifestyle-associated interventions are compelling in geroscience because they are scalable, comparatively low risk, and clinically relevant, but their effects on biological aging cannot be evaluated rigorously without standardized molecular and functional assessment. A translational investigative framework should select among cellular senescence, immune remodeling, mitochondrial function, oxidative stress, metabolic regulation, proteostasis/autophagy, and epigenetic aging, and anchor the selected molecular measures to whole-organism functional outcomes. Within this framework, redox- and mitochondria-oriented interventions, including selected phytochemicals, should be evaluated as mechanistically plausible but still experimental modulators of aging-related pathways, using standardized molecular and functional endpoints rather than broad antioxidant or anti-aging claims.

In this context, biomarker-guided geroscience offers a practical path forward. By combining mechanistic relevance, analytical feasibility, clinical interpretability, and functional endpoints, human studies can move beyond generalized anti-aging rhetoric toward testable strategies for preserving resilience, healthspan, and functional independence.

## Figures and Tables

**Table 1:** Aging mechanisms and corresponding biomarker domains.

Aging Mechanism	Candidate Biomarkers	Sample Type	Assay Method	Strengths	Limitations	Multicenter Suitability	Ref.
Epigenetic aging	DNAm PhenoAge; GrimAge/GrimAge2; DunedinPACE	Whole-blood DNA	Illumina methylation arrays	Integrative; outcome-associated	Different constructs and CpGs; batch/cell-composition effects; not validated lifespan surrogates	Moderate to high with centralized processing	[19,20,21,22,24]
Cellular-senescence-associated measures	p16^INK4a^; p21^CIP1/WAF1^; complementary cell/tissue features; SASP-related proteins	Defined cells/tissue; PBTL/PBMC RNA; plasma	qPCR; imaging; immunoassays	Mechanistically relevant	No single specific marker; blood does not quantify organ burden	Moderate; cellular/tissue modules	[5,27,30,31,32]
Systemic inflammation/stress	hsCRP; IL-6; sTNFR1/2; GDF-15	Serum/plasma	Immuno-turbidimetric; immunoassays	Feasible; clinically familiar	Not senescence-specific; GDF-15 is an integrated stress signal	High	[1,10,11,12,33,34,35,36]
Oxidative stress	F2-isoprostanes; 8-OHdG	Plasma or urine; matrix prespecified	Validated isotope-dilution GC-MS or LC-MS/MS	Mechanistically relevant	Ex vivo oxidation; urine dilution; avoid nonspecific colorimetric/TBARS assays	Moderate	[14,15,16,17,18,37,38,39]
Mitochondrial dysfunction	mtDNA abundance; respiration; mtDNA sequence integrity when indicated	Blood; PBMCs; tissue-specific substudies	qPCR; respirometry; 31P-MRS; targeted sequencing	Strong biological rationale	Content is not function; tissue/cell composition and platelet effects	Low to moderate; targeted modules	[13,28,40,41,42]
Metabolic dysregulation	Glucose; insulin; HOMA-IR; HbA1c; lipids	Serum/plasma	Standard clinical chemistry	Highly feasible; clinically interpretable	Not aging-specific	High	[43,44,45]
Proteomic/metabolomic aging	Proteomic clocks; metabolomic signatures	Plasma/serum	Olink; SomaScan; NMR	High-dimensional integration	Platform dependence; normalization burden	Moderate	[23,46,47,48,49,50,51,52]
Functional decline (clinical outcomes)	Gait speed; grip strength; frailty index; SPPB	Clinical performance	Standardized functional testing	Clinically meaningful	Operator training required	High	[2,25,53,54,55,56]
Immune remodeling	Naive/memory subsets; activation phenotype	Blood/PBMCs	Flow cytometry	Cell-resolved	Infection, cell composition, and processing; not a senescence assay	Moderate; centralized protocol	[9,28]
Proteostasis/autophagy; regenerative reserve	Process-specific functional assays; tissue-dependent regeneration measures	Cells/tissue in substudies	Validated functional/flux assays	Mechanistic depth	Static protein abundance is not flux; no universal circulating stem-cell-exhaustion marker	Low; exploratory	[9,27]

SASP—senescence-associated secretory phenotype, PBTL—peripheral blood T-lymphocytes, PBMC—peripheral blood mononuclear cells, qPCR—quantitative polymerase chain reaction, hsCRP—high-sensitivity C-reactive protein, IL-6—interleukin-6, GDF-15—growth differentiation factor 15, 8-OHdG—8-hydroxy-2′-deoxyguanosine. GC-MS—gas chromatography-mass spectrometry, LC-MS/MS—liquid chromatography-tandem mass spectrometry, mtDNA—mitochondrial DNA, 31P-MRS—phosphorus-31 magnetic resonance spectroscopy, HOMA-IR—homeostatic model assessment of insulin resistance, HbA1c—hemoglobin A1c (glycated hemoglobin), NMR—nuclear magnetic resonance, SPPB—short physical performance battery. sTNFR1/2—soluble tumor necrosis factor receptors 1/2; TBARS—thiobarbituric-acid-reactive substances. Functional measures are clinical outcome assessments under BEST; the remaining rows concern molecular/cellular biomarker domains.

**Table 2:** Lifestyle interventions: a mechanism-based roadmap for biomarker selection.

Intervention	Target Process/Pathway	Main Readout(s)	Complementary/Orthogonal Readout(s)	Functional Outcomes	Response Window and Interpretation	Ref.
Exercise	Mitochondrial biogenesis and function; AMPK signaling; insulin sensitivity	Mitochondrial hypothesis: respiratory capacity in defined PBMCs or muscle tissue	Citrate synthase activity in the same sample; fasting insulin/HOMA-IR for metabolic context	Aerobic fitness; grip strength; gait speed (select by training type)	Test improved respiration/function at 3–6 months; content need not increase. Control recent exercise, cell composition and adherence. PBMC changes do not establish improved **muscle** mitochondrial function.	[9,28,54,63,64,65,69,70]
Dietary optimization	Metabolic regulation; insulin sensitivity; inflammatory signaling	Fasting insulin/HOMA-IR; LDL cholesterol when lipid lowering is the target	hsCRP for inflammatory context; body composition for adiposity/lean mass	Grip strength or SPPB in older, functionally at-risk participants	Test lower HOMA-IR/LDL at 4–6 weeks; reassess sustained change and function at 3–6 months. Account for baseline values, dietary adherence, weight change and medication.	[44,71]
Caloric restriction/time-restricted eating	Nutrient sensing (AMPK–mTOR); metabolic flexibility; autophagy hypothesis	Fasting insulin/HOMA-IR; a dynamic insulin-sensitivity test in a substudy	Body composition; HbA1c in dysglycemia. A CR-specific senescence module may be added (Section 4.2).	Grip strength or SPPB; monitor loss of function	Test lower insulin/HOMA-IR at 4–6 weeks and preserved function at 3–6 months. Record energy intake and eating window separately; do not extrapolate CR findings to TRE.	[9,30,45]
Sleep/circadian intervention	Circadian regulation; inflammatory and metabolic signaling	For an inflammatory hypothesis: resting IL-6 or hsCRP	Actigraphy: sleep timing and regularity (exposure); diurnal cortisol for a stress-axis hypothesis	Daytime sleepiness or cognition (validated assessment)	Assess sleep at 4–6 weeks; inflammation and function at 3–6 months. A fall in resting IL-6/hsCRP is not assured. Control sampling time, sleep apnea and sedatives.	[72,73]
Stress reduction	Autonomic and stress-axis regulation; inflammatory signaling	Resting HRV or diurnal cortisol profile (saliva; prespecify the metric)	Resting IL-6 or hsCRP; do not equate resting and stimulated cytokine assays	Daily function or cognition (validated assessment)	Assess at 4–6 weeks and 3–6 months. Prespecify HRV/cortisol direction from the hypothesis; circulating inflammation may not fall. Standardize breathing/posture, sampling time and medication.	[72,73,74,75]
Nutraceutical add-on	Compound-specific; example: oxidative damage and mitochondrial function	Redox hypothesis: F2-isoprostanes by validated GC-MS or LC-MS/MS	PBMC respiratory capacity if a mitochondrial mechanism is also hypothesized	Grip strength or 6MWT when physical function is the stated target	Test lower oxidative damage with preserved/improved function. A 3–6-month assessment requires compound-specific justification. Control formulation, dose, exposure, interactions and safety; preclinical activity is not human efficacy.	[28,38,69,76,77,78,79]

hsCRP—high-sensitivity C-reactive protein; IL-6—interleukin-6; HbA1c—glycated hemoglobin; HRV—heart rate variability; PBMC—peripheral blood mononuclear cell; HOMA-IR—homeostatic model assessment of insulin resistance; LDL—low-density lipoprotein; AMPK—AMP-activated protein kinase; mTOR—mechanistic target of rapamycin; CR—caloric restriction; TRE—time-restricted eating; SPPB—Short Physical Performance Battery; 6MWT—6-min walk test; GC-MS—gas chromatography–mass spectrometry; LC-MS/MS—liquid chromatography–tandem mass spectrometry. Select one or two main readouts and a relevant functional outcome; entries are alternatives, not mandatory panels. Complementary measures provide orthogonal support or exposure/context information, as identified, not proof of pathway engagement. Response windows are illustrative protocol-design choices, assessed from baseline against a comparator, not validated minimum response times.

**Table 3:** Common measurements and mechanism-specific modules for human aging studies.

Biomarker Category	Common Core/Selected Secondary Endpoint	Mechanism-Specific or Exploratory Module	Illustrative Assessment Windows	Interpretation Caveats	Ref.
Epigenetic	No mandatory clock. Prespecify DNAm PhenoAge or GrimAge2 for phenotype/risk, or DunedinPACE for pace (secondary)	Additional clocks only for prespecified comparison; tissue-specific studies	Baseline and 6–12 months; longer follow-up when justified	Different constructs; no literal lifespan interpretation	[19,20,21,22,24,60,66]
Senescence	Not a universal core measure	Complementary cellular/tissue features; p16^INK4a^ in defined cells; selected SASP factors	Baseline and 3–6 months; extend to 12 months as justified	Blood proxies do not quantify tissue senescence; combine independent features	[5,27,30,31,32,54,63]
Inflammatory/stress-associated	hsCRP; selected IL-6, GDF-15 and/or sTNFR1 or sTNFR2 according to objective	Expanded cytokine/chemokine panels	Baseline; 4–6 weeks; 3 or 6 months; 12 months	Infection, adiposity, renal function, exercise and medication; not tissue-specific	[1,10,11,33,34,35,36,62]
Oxidative stress	Not a universal core measure	One chemically specific MS-validated redox marker; expanded panel if justified	Baseline and 3–6 months; earlier if mechanism warrants	Matrix, handling, ex vivo oxidation and urine dilution	[38,39]
Mitochondrial	Not a universal core measure	Respiration plus content-related measure in the same cells; tissue assay where feasible	Baseline and 3–6 months; 12 months for persistence	Content, sequence integrity and function differ; control cell composition	[28,40,41,42]
Metabolic	Glucose; HbA1c; lipids; fasting insulin/HOMA-IR when indicated	Acylcarnitines; amino acid panels	Baseline; 4–6 weeks for glucose/insulin; 3–6 months for sustained response; 12 months	Clinically useful but not aging-specific	[36,44,45]
Functional clinical outcomes	Grip strength; gait speed; SPPB; frailty index or 6MWT according to population	Cardiorespiratory fitness; cognitive testing; balance	Baseline; 3 or 6 months; 12 months	Training and protocol consistency matter	[2,25,53,54,55,56]

DNAm—DNA methylation, SASP—senescence-associated secretory phenotype, hsCRP—high-sensitivity C-reactive protein, IL-6—interleukin-6, GDF-15—growth differentiation factor 15, HOMA-IR—homeostatic model assessment of insulin resistance, HbA1c—hemoglobin A1c (glycated hemoglobin), SPPB—short physical performance battery, 6MWT—6-min walk test. sTNFR1/2—soluble tumor necrosis factor receptors 1/2. Timing is illustrative, not a universal minimum detectable-response interval. The endpoint, assay, population, and comparator determine the primary contrast.

**Table 4:** Practical considerations for multicenter biomarker studies.

Challenge	Impact on Data Quality	Recommended Solution	Ref.
Time-of-day variation	Artificial between-person and between-site differences	Fix collection windows and record timing	[10,11,72,73]
Recent exercise or acute illness	Distorts inflammatory and metabolic markers	Use pre-visit restrictions and illness screening	[1,10,11,64,65]
Sample handling delays	Degrades sensitive analytes	Harmonize processing times and storage SOPs	[10,11,37,48,49,51]
Batch effects	False biological signals in omics data	Randomize batches and centralize QC	[19,20,24,48,49,51]
Platform variability	Reduced comparability across sites	Use centralized laboratories when possible	[48,49,51]
Medication and comorbidity confounding	Misleading biological interpretation	Collect detailed covariates and prespecify adjustments	[1,25,33,34,35,44]
Overly broad biomarker panels	Cost inflation and analytic noise	Separate core from exploratory endpoints	[3,24,25,47,50,52]

## Data Availability

No new data were created or analyzed in this study. Data sharing is not applicable to this article.

## Abbreviations

The following abbreviations are used in this manuscript:

31P-MRS	phosphorus-31 magnetic resonance spectroscopy
6MWT	6-min walk test
8-OHdG	8-hydroxy-2′-deoxyguanosine
AI	artificial intelligence
BEST	Biomarkers, EndpointS, and other Tools
BRISQ	Biospecimen reporting for improved study quality
CCL	C-C motif chemokine ligand
CSF	cerebrospinal fluid
CXCL	C-X-C motif chemokine ligand
DNAm	DNA methylation
EDTA	ethylenediaminetetraacetic acid
FDA	U.S. Food and Drug Administration
FEV1	forced expiratory volume in one second
FVC	forced vital capacity
GC-MS	gas chromatography-mass spectrometry
GDF-15	growth differentiation factor 15
HbA1c	hemoglobin A1c (glycated hemoglobin)
HO-1/HSP32	heme oxygenase-1
HOMA-IR	homeostatic model assessment of insulin resistance
HRV	heart rate variability
hsCRP	high-sensitivity C-reactive protein
IGF-1	insulin-like growth factor 1
IL	Interleukin
LC-MS/MS	liquid chromatography-tandem mass spectrometry
LDL	low-density lipoprotein
MCP	monocyte chemoattractant protein
mtDNA	mitochondrial DNA
MTMC	multi-threat medical countermeasures
NAD	nicotinamide adenine dinucleotide (oxidized form)
NADH	nicotinamide adenine dinucleotide (reduced form)
NIH	National Institutes of Health
NMR	nuclear magnetic resonance
PAI-1	plasminogen activator inhibitor 1
PathStAR	pathology-based structural aging rate
PBMC	peripheral blood mononuclear cells
PBTL	peripheral blood T-lymphocytes
PGT	Pyunkang-Tang
qPCR	quantitative polymerase chain reaction
ROS	reactive oxygen species
SASP	senescence-associated secretory phenotype
SPPB	short physical performance battery
SPREC	standard preanalytical code
TAME	targeting aging with metformin
TNF-α	tumor necrosis factor α
AMPK	AMP-activated protein kinase
mTOR	mechanistic target of rapamycin
sTNFR1/2	soluble tumor necrosis factor receptors 1/2
TBARS	thiobarbituric-acid-reactive substances
CR	caloric restriction
TRE	time-restricted eating
